# Determining the Efficacy of a Hybridizing Agent in Wheat (*Triticum aestivum* L.)

**DOI:** 10.1038/s41598-019-56664-9

**Published:** 2019-12-27

**Authors:** Amanda C. Easterly, Walter W. Stroup, Nicholas Garst, Vikas Belamkar, Jean-Benoit Sarazin, Thierry Moittié, Amir M. H. Ibrahim, Jackie C. Rudd, Edward Souza, P. Stephen Baenziger

**Affiliations:** 1 Department of Agronomy and Horticulture, University of Nebraska, Lincoln, NE 68583-0915 United States; 2 Department of Statistics, University of Nebraska, Lincoln, NE 68583-0963 United States; 3 Asur Plant Breeding, Estrées-Saint-Denis, Picardy, France; 4 Department of Soil and Crop Sciences, Texas A&M University, College Station, TX 77843 United States; 5 Texas AgriLife Research and Extension Center at Amarillo, Amarillo, TX 79106 United States; 6 BASF, Beaver Crossing, NE 68313 United States

**Keywords:** Plant breeding, Plant genetics

## Abstract

Hybrid wheat (*Triticum* spp.) has the potential to boost yields and enhance production under changing climates to feed the growing global population. Production of hybrid wheat seed relies on male sterility, the blocking of pollen production, to prevent self-pollination. One method of preventing self-pollination in the female plants is to apply a chemical hybridizing agent (CHA). However, some combinations of CHA and genotypes have lower levels of sterility, resulting in decreased hybrid purity. Differences in CHA efficacy are a challenge in producing hybrid wheat lines for commercial and experimental use. Our primary research questions were to estimate the levels of sterility for wheat genotypes treated with a CHA and determine the best way to analyze differences. We applied the CHA sintofen (1-(4-chlorphyl)-1,4-dihydro-5-(2-methoxyethoxy)-4-oxocinnoline-3-carboxylic acid; Croisor 100) to 27 genotypes in replicate. After spraying, we counted seed in bagged female heads to evaluate CHA efficacy and CHA-by-genotype interaction. Using logit and probit models with a threshold of 7 seeds, we found differences among genotypes in 2015. Sterility was higher in 2016 and fewer genotypic differences were found. When CHA-induced sterilization is less uniform as in 2015, zero-inflated and hurdle count models were superior to standard mixed models. These models calculate mean seed number and fit data with limit-bounded scales collected by agronomists and plant breeders to compare genotypic differences. These analyses can assist in selecting parents and identifying where additional optimization of CHA application needs to occur. There is little work in the literature examining the relationship between CHAs and genotypes, making this work fundamental to the future of hybrid wheat breeding.

## Introduction

Increasing winter wheat (*Triticum aestivum* L.) grain yield and developing cultivars better adapted to climactic variability is crucial for agricultural productivity and food security. Developing hybrid wheat may be a way to address these goals^1^. Hybrid cultivars of crop plants are first generation seed of a cross (hybrid) of two or more inbred parents. Excellent hybrid cultivars exhibit heterosis, in which the performance of the crop for a trait of interest exceeds the performance for that trait in the individual inbred parents. Hybrids revolutionized maize (*Zea mays* L.) and sorghum (*Sorghum bicolor* L. Moench) breeding^2,3^ and production. Since the adoption of hybrid maize, yields in the United States increased by more than five-fold in the 20^th^ century, much of which is attributed to heterosis and breeding for hybrid performance^3,4^. Yet wheat breeders remain skeptical of the viability of hybrid wheat^5^. Wheat is a self-pollinated crop with perfect flowers (anthers and stigma are in the same flower) as compared to maize with imperfect flowers where the male and female organs are separated in the tassel and ears. For sorghum, hybrids are produced using genetic and cytoplasmic male sterility. In wheat, sterility systems like these are complex^6^. Therefore, a limitation to evaluating hybrid wheat performance is efficient and reliable hybrid seed production^5,6^.

Chemically induced male sterility is used as a method in wheat to force cross-pollination, resulting in hybrid seed^5,6^. These chemical hybridizing agents (CHAs) induce male sterility through suppression of pollen formation^6^. However, CHAs can be incomplete in their function for a variety of reasons, namely that CHAs require the application to be well-timed to the crop development stage and at appropriate dosage for the genotype^7^. Despite these challenges, CHAs remain one of the simplest means to produce hybrid wheat cultivars for testing if high levels of sterility can be obtained^6^. Evaluating the efficacy of the CHA is a necessary step for experimental and commercial hybrid seed production to ensure high levels of hybridity and purity during seed production. Quantifying the efficacy of a CHA with respect to genotypic differences will enable parental selection for future hybrid production and evaluation.

Modern statistics has roots in agricultural research^8–10^. In recent years, the approaches to analyze many types of data and available computing capabilities have improved. The methodology commonly used in agricultural fields has not always kept pace with these statistical advances^11^. Classical statistics considers primarily quantitative data in simple designs. The observations are generally assumed to have a continuous, Gaussian (or normal) distribution^11^. However, in agronomy and other disciplines, operating within the assumptions of normality is not always possible. For a number of agronomically important questions, experiments may have responses that are counts, proportions, frequencies, binary categories, or rankings. These types of data do not neatly fit into statistical frameworks that assume normality^11,12^. For example, in hybrid wheat research the female parents are treated with a CHA. To measure efficacy, individual heads are isolated under pollen-impermeable bags to test if adequate sterility was achieved^13^. These data are reported as a count of the number of seeds on each isolated head. Ideally, the seed count is close to zero—where a count of zero seeds indicates total sterility. These data are problematic for statistical analysis because of the non-Gaussian distribution with a high frequency of zero counts. How best to model this sterility data? Historically, logarithmic or square root data transformation approximates for the normal distribution were used in analysis^11^. A key constraint with data transformations is that the estimates are not easily interpreted on the transformed scale and when back-transformed can result in estimates that are nonsensical (i.e. negative counts). Furthermore, when normally-distributed random effects in the model are considered, transformations distort the estimates of random effects and may result in misleading conclusions. In experiments such as these, random effects would include terms for variation within plots, design factors such as blocks or replications that are of secondary interest to the fixed effect of genotype. Others have estimated the proportion of the bagged heads that have an acceptable number of seeds. Based on this proportion, sterility is accepted to have occurred if a certain threshold, defined as a number of seeds, is met^13^. For an initial screening of the material, the use of logit or probit models can be simple and beneficial. The constraint in using binary results and logit or probit models (i.e. if the CHA is effective or ineffective) of adequate sterility is that they limit the ways in which a plant breeder can use the data. More importantly, they may not be valid because the number of seeds per fertile head can vary greatly across and within genotypes for hard winter wheat and so modeling the counts of seed directly is ideal.

Standard models for count data use Poisson or negative binomial distributions. However, these distributions cannot adequately account for data with large frequencies of zeros and thus result in inflated variances and overdispersion^14–16^. Models that use mixture distributions such as zero-inflated and hurdle models, are a way to overcome this poor fit. Ridout *et al*.^14^, Chin and Quddus^17^, and Stroup^15^ provide detailed reviews of these distributions. The equations below show the probability density functions for zero-inflated model (Eq. 1) and the hurdle model (Eq. 2) where y is the observed count of seeds for a head. Zero-inflated and hurdle models utilize the probability distribution functions for either Poisson or Negative Binomial distributions when the seed count is greater than or equal to zero. The two models differ in their treatment of values of zero.

Zero-inflated models assign a probability of *π* (called the inflation probability or mixture weight) for the wheat head to be totally sterile due to the CHA (i.e. have a seed count of zero) but also allow for the possibility that a situation with no seeds may arise for other reasons such as random kernel abortion^14,16,18^.

1 $$\Pr \{Y=y\}=\{\begin{array}{ll}\pi +(1-\pi )\,f(0) & for\,y=0\\ (1-\pi )\,f(y) & for\,y=1,2,\ldots \end{array}$$

For hurdle models, the inflation probability (*π*) is mutually exclusive from the probability of obtaining any other count (1 − *π*). In the case of modeling sterility data for hybrid wheat, a value of zero is observed at a proportion, *π*, if and only if the CHA has been fully effective for that head. It thus assumes that a count of zero seeds is due strictly to the effects of the CHA and not any other outside processes.

2 $$\Pr \{Y=y\}=\{\begin{array}{cc}\pi  & for\,y=0\\ (1-\pi )\frac{f(y)}{1-f(0)} & for\,y=1,2,\ldots \end{array}$$

Zero-inflated and hurdle models are commonly used in biomedical, epidemiological, manufacturing, and ecological research and are well-established in those disciplines^14,19–22^. Biologically, a hurdle model is more likely to reflect the way in which the counts arise for sterility data, because wheat will generally always form some seeds on the head if not treated by a CHA. What is unknown is if these models better suit data for wheat treated with a contact-based CHA, and how these models compare to other statistical modeling strategies.

Other ways to address data such as these may include probit and logit models as used in toxicology (i.e. use of probit models to determine LD50 estimates for exposure to chemicals). Probit and logit models can be used as a first step for evaluating sterility data using an appropriate threshold based on minimum desired sterility. If all genotypes meet the desired threshold, then researchers can be confident in the level of hybrid purity in their seed. The constraint, however, is that the data is collected as counts, not proportions. Because probit and logit models are for modeling proportions, their use for this type of data is technically inappropriate. An additional constraint to the use of logit and probit models results from the natural variability within and among genotypes for total possible number of seeds per head. In addition, fertile seed is simple to count whereas sterile florets do not form seed and it is difficult to determine how many total florets could have formed. Thus, it is preferred to model the fertile seed directly and use it to determine how much sterility was achieved.

Our goal was to determine the efficacy of a CHA on numerous wheat genotypes using the most appropriate statistical approaches. Specifically, we wanted to determine the proportion of sterility from CHA application, seed count if not totally sterile, and genotype interactions to the treatment with CHA. Few, if any, studies have tested CHA efficacy as a function of genotypic differences or determined statistical modeling strategies. This study is part of a multi-institutional research program to produce and evaluate experimental hybrids in wheat and focuses on the hybrid seed production aspect of that project.

## Materials and Methods

### Germplasm and experimental design

Seed production of hybrid wheat was conducted using crossing blocks separated by 9 meters of triticale (*x Triticosecale*) as both a physical and genetic buffer against unplanned cross-pollination. A total of 27 winter wheat genotypes were tested as female parents across 2015 and 2016. Of these 27 genotypes, 25 were constant across both years. To optimize genetic diversity, the genotypes included cultivars and elite breeding lines from the University of Nebraska-Lincoln and Texas A&M University wheat breeding programs (Table 1). The female genotypes were planted in a randomized complete block design and sprayed with the CHA, Croisor 100 (active ingredient sintofen; 1-(4-chlorophenyl)-5-(2-methoxyethoxy)-4-oxo-1,4-dihydrocinnoline-3-carboxylic acid) (Asur Plant Breeding, Estrées-Saint-Denis, France). The sterilized females were pollinated by unsterilized male rows of a single genotype that surrounded the sterilized female block (Fig. S1). The female lines were planted in plots of five rows spaced 23 cm apart and 3.2 meters long at a density of roughly 650 seeds meter^−2^. The high seeding rate of females was intentional in order to decrease the number of late tillers and thus better synchronize female head development for a more uniform growth stage among tillers at chemical application. On the contrary, male lines were planted at a lower seeding rate (approximately 150 seeds meter^−2^) in order to have higher number of tillers to increase the window of pollination. The trials were located at the University of Nebraska’s Agricultural Research and Development Center (ARDC) near Mead, NE, USA.

**Table 1:** Genotypes included in sterility assay in 2015 and 2016.

Entry Number	Name	Years Included	Breeding Program of Origin
1	Freeman	2015, 2016	UNL
2	Goodstreak	2015, 2016	UNL
3	Harry	2016	UNL
4	LCH13NEDH-11-24	2015, 2016	UNL
5	NE07531	2015, 2016	UNL
6	NE09517-1	2015, 2016	UNL
7	Ruth	2015, 2016	UNL
8	NE10683	2015, 2016	UNL
9	Overland	2015, 2016	UNL
10	Panhandle	2015, 2016	UNL
11	PSB13NEDH-15-58W	2015, 2016	UNL
12	Robidoux	2015, 2016	UNL
13	Settler CL	2015, 2016	UNL
14	TX09D1172	2015, 2016	TAMU
15	TX10D2063	2015, 2016	TAMU
16	TX10D2230	2015, 2016	TAMU
17	TX10D2363	2015, 2016	TAMU
18	TX11D3008	2015, 2016	TAMU
19	TX11D3026	2015, 2016	TAMU
20	TX11D3049	2015, 2016	TAMU
21	TX11D3112	2015, 2016	TAMU
22	TX11D3129	2015, 2016	TAMU
23	TX12M4004	2015, 2016	TAMU
24	TX12M4063	2015, 2016	TAMU
25	TX12M4065	2015, 2016	TAMU
26	Wesley	2015, 2016	UNL
27	NE10478-1	2015	UNL

^a^UNL, University of Nebraska-Lincoln; ^b^TAMU, Texas A&M University.

In the plots of each female genotype, five heads were covered with pollen impermeable bags (Size 117, Lawson Bag Company, Northfield, IL, USA) roughly 3 weeks post-CHA application in four crossing blocks in each year (described in detail below). Thus, there were four experimental units per genotype per year and five sampling units per experimental unit. Bags were secured to the plant using bamboo stakes and laboratory tape to keep them attached to the plant through the rest of the growing season. This process allowed for the head to develop normally but prevented pollination from the adjacent male plots and reduced loss of bagged heads due to severe weather. After the grain filling period and prior to plot harvest, each bagged head was harvested, threshed individually, and the number of seeds recorded. If the CHA was fully effective, the head had empty florets where seeds failed to form due to complete sterilization. If seeds were present, it was assumed that the CHA had not been completely effective and the seed formed from self-pollination. Due to weather conditions, particularly the strong winds of the Great Plains, some bags were lost and those observations affected the balance of the dataset.

### Chemical hybridizing agent and application

Plots were treated with Croisor 100 at a rate recommended by the manufacturer. ChemSurf 90 (active ingredients: glycerol, sodium xylene sulfonate, alkyl phenol ethoxylate) was used as a surfactant at a rate of 0.3 ml m^−2^ (United Suppliers, Eldora, IA, USA). Water was added to the mix for a total spray volume of 30 ml m^−2^ and applied using a 3.5 m wide hooded sprayer at 234 kPa. The application timing was at the average plant growth stage of 30-33^23^. Immature heads in the stem were between 1.5 and 1.8 cm, per the CHA manufacturer’s recommendation. Application dates differed based on environmental conditions in each year: 12 May 2015 and 22 April 2016. Genotypes were staged frequently and individually by genotype to ensure that the immature heads were at the proper size for treatment with the CHA. While the germplasm showed some variation for gape date (number of Julian days to 50% gaping of the sterile head) later in the season (Table S1), nearly every genotype was within the optimal CHA treatment window at the same time.

### Probit and logit models

Using a count of seven seeds or fewer as the cutoff value, each observed head was classified as either a success (“1”) or failure (“0”). The cutoff value was selected because the mean number of total seeds on a healthy head of hard winter wheat in the Great Plains is around 30. Thus, seven seeds correspond to a sterility level of about 75% and that value would be acceptable for experimental hybrid seed production. The PROC GLIMMIX and DATA steps of SAS 9.4 software (SAS Institute, Inc., Cary, NC, USA) were used to evaluate logit and probit threshold models. The linear predictor was used as follows with a log link:

3 $${\eta }_{ijk}={\alpha }_{i}+{b}_{j}+{(\alpha b)}_{ijk}$$

Where *η*_*ijk*_ is the frequency of observing seven seeds or fewer for head (*αb*)_*ijk*_ of genotype *α*_*i*_ in block b_*j*_. The random *b*_*j*_ and (*αb*)_*ijk*_ effects were assumed independent and identically distributed (i.i.d.) normally with a mean of zero and variances of ${\sigma }_{b}^{2}$ and ${\sigma }_{ab}^{2}$, respectively. Genotypic means ($\widehat{{\alpha }_{i}}$) and confidence intervals for the genotypic means were obtained as inverse link estimates for each genotype in each year.

The models were fit using adaptive Gauss-Hermite Quadrature^24^. Model fit was evaluated across the logit and probit models using the corrected Akaike’s Information Criterion (AICc), Bayesian Information Criterion (BIC), and −2 Log Likelihood. The more conservative AICc fit statistic was examined rather than the standard AIC due to relatively small sample sizes^25^.

### Statistical count models

Counts of seed per bagged head for each genotype were analyzed by mixed effects analysis using PROC UNIVARIATE, PROC GLIMMIX, and PROC NLMIXED procedures in SAS 9.4 software. Transformed response variables were incorporated into the dataset using a DATA step in SAS 9.4 software to evaluate the efficacy of transformed data with a normal approximation (SAS Institute, Inc., Cary, NC, USA). These transformations include logarithmic, square root, and exponential transformations as follows:

$${\textstyle \text{``}}log\,count{\textstyle \text{''}}=\,\log \,(seed\,count+1)$$

$${\textstyle \text{``}}sqrt\,count{\textstyle \text{''}}=\sqrt{seed\,count+3/8}$$

$${\textstyle \text{``}}exp\,count{\textstyle \text{''}}={(seedcount)}^{2/3}$$

Initial analyses were done by year, and estimates for seed count were modelled as in Eq. 3, this time letting *α*_*i*_ be the estimated seed count for genotype *i* rather than a binary response.

Distributions, GLMM estimators, and appropriate link functions for Poisson and negative binomial models are described in Stroup^15^ and given in Table 2. Zero-inflated negative binomial models were coded into PROC NLMIXED as shown in Eq. 1. Hurdle negative binomial models also were coded into PROC NLMIXED as shown in Eq. 2. The first run for each year allowed an inflation probability (π) and lambda (λ) terms specific to each genotype, where lambda is the mean seed count for a genotype after treatment with the CHA. The second run included only a single inflation probability for the experiment as a whole with individual lambda (mean count) terms for the Negative Binomial part of the function.

**Table 2:** Estimation methods used in each model tested.

Model	Estimation Technique	Link Function
Gaussian (Normal Approximation)	Restricted Maximum Likelihood	Identity
Poisson	Maximum Likelihood using the Laplace method	Logarithmic
Negative Binomial	Maximum Likelihood using the Laplace method	Logarithmic
Log-transformed with normal approximation (LT)	Restricted Maximum Likelihood	Identity with back-transformation
Square-root-transformed with normal approximation (ST)	Restricted Maximum Likelihood	Identity with back-transformation
Exponential transformation with normal approximation (ET)	Restricted Maximum Likelihood	Identity with back-transformation
Zero-inflated Negative Binomial (ZINB)	Maximum Likelihood using the Laplace method	Logarithmic (Negative binomial process) and logit (inflation probability)
Hurdle Negative Binomial (HNB)	Maximum Likelihood using the Laplace method	Logarithmic (Negative binomial process) and logit (inflation probability)

Estimates of genotypic means were obtained using LSMEANS statements in PROC GLIMMIX and ESTIMATE statements in PROC NLMIXED. These estimates are expressed on the data scale using the inverse log link function. The AICc, BIC fit statistics were used to identify best model fit. Pearson Chi-Square values (χ^2^/d.f.) were used to asses overdispersion in the Poisson models. Data also were analyzed across both 2015 and 2016, and included a fixed effect for year variation between the two years of testing. However, the models rarely converged and this limited the ability to obtain credible results from the combined analysis. A follow up study is underway to evaluate the results from a smaller number of genotypes across years and evaluate the genotype-by-year interaction in more detail.

### Simulated data and power analysis

Data were simulated for two sets of 500 experiments from a zero-inflated negative binomial process using DATA steps for Bernoulli and negative binomial random variables within SAS 9.4 software. The selected parameters for each genotypic count mean and inflation probability are outlined in the supplementary online resource materials.

For each simulated experiment, Gaussian, Poisson, negative binomial and transformed data models were run and estimates of seed count obtained as described for the experimental data above, with the exception that subsampling effects were left out for computational efficiency. The results for including the subsampling term were nearly identical to the results without in preliminary runs of the simulations and for experimental designs such as this, the dropping of subsampling effects is documented in the literature^26^. The estimates were written out and the lower and upper confidence bounds were transformed to the data scale as needed for the estimated seed counts. Using the set parameters from which the data were simulated, the confidence intervals were tested for coverage—to see if the parameter was contained within the interval. Coverage was recorded as a binary variable, 1 for true and 0 for false, where ‘false’ indicated that the true mean was not in the 95% confidence interval. The mean coverage was calculated for each model tested across the 500 experiments in duplicate for a total of 1000 experiments. The empirical coverage proportion indicates how well the models tested are able to estimate the mean count for each genotype if the counts do arise from a zero-inflated or hurdle process.

## Results

### Summary of raw data and transformed variables

**Figure 1:**
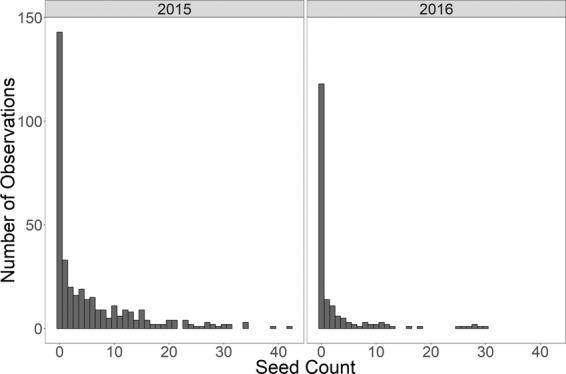
Histograms showing seed count data in 2015 (left) and 2016 (right) showing high frequency of zero seeds.

The mean number of seeds per observation in 2015 was 5.7 seeds head^−1^, which was reduced in 2016 to 2.6 seeds head^−1^. The 2016 value was less than half of the previous year and had a smaller standard deviation (Table 3). The improvement is attributed to greater familiarity and precision in using the CHA during the second year. In both years, the counts were skewed (1.8 and 3.2 in 2015 and 2016, respectively) towards zero providing additional evidence that the assumption of the normal approximation is inappropriate. Figure 1 shows the distribution of the raw data across all genotypes. Detailed summary statistics including number of observations, mean, median, and mode for both years are included in Table 3.

**Table 3:** Summary statistics for 2015 and 2016 hybrid wheat sterility data.

Trait	2015	2016
Number of observations	371	182
Mean seed count	5.7	2.6
Variance	65.1	35.8
Standard deviation of seed count	8.1	6.0
Proportion of observations with 0 seeds	0.38	0.64
Skewness	1.8	3.2
Median seed count	2	0
Mode seed count	0	0
Minimum seed count	0	0
Maximum seed count	42	30

Based on quantile distributions and the observed proportion of zero seeds, better sterility was achieved in the trial in 2016 where 64% of the heads had a count of zero than in the trial 2015 where the percentage of observed heads with a count of zero was 38% (Table 3).

### Logit and probit model fit statistics and genotypic differences

As an initial test, logit and probit models assuming a count of seven or fewer seeds as a success were evaluated for individual years. The fits were nearly identical for both models within the same year. In 2015, the probit model had an AICc of 405.43 and the logit model had an AICc of 409.81 (Table S2). The Type III tests for fixed effects of genotype were calculated and there were differences among the genotypes for frequency of meeting the threshold at alpha = 0.05 (Table 4). Thus, the 2015 data showed that there were significant differences among genotypes for probability of being sterile using a threshold of seven seeds or fewer. Least squares means were obtained for each genotype using the probit model (Table S3). Least squares means were also obtained for each genotype in 2016 using the logit model (Table S4).

**Table 4:** Type III tests for genotype effects in threshold models assuming a threshold of seven seeds or fewer as acceptable.

Year	Threshold Model	F	Pr > F
2015	Logit	1.66	0.0257
	Probit	1.78	0.0136
2016	Logit	0.40	0.9914
	Probit	0.63	0.7997

In 2015, ‘TX12M4065’ had the lowest estimate at 0.32, indicating that a count of seven seeds or fewer was only observed 32% of the time. In contrast, ‘Ruth’ and ‘LCH13NEDH-11-24’ had an estimate of 1.00 meaning they were completely sterile, though some results had p-values greater than 0.05 and the confidence in the result is thus lower. Overall, 12 of the 26 genotypes tested in 2015 met the threshold of sterility at a minimum frequency of 0.90. Of the remaining genotypes, seven had mean estimates over 0.80, with the remaining eight genotypes ranging from 0.32 to 0.76.

In 2016, the logit model had an AICc value of 77.95 whereas the probit model’s AICc was 77.51 (Table S2). The Type III tests for fixed effects of genotype in 2016 revealed that there was no significant variation of frequency for meeting the threshold among genotypes in 2016 (Table 4). Simply put, the 2016 data had overall higher sterility and as a result, there were no significant differences among genotypes and every genotype was effectively sterilized and had less than seven seeds per head. In examining the genotype-specific means, the mean proportion was always greater than 70%, with most genotypes exhibiting seven seeds or fewer all of the time (Table S4).

### Count model fit statistics and comparisons

The next step was to identify the best analysis for situations in which the logit and probit approach identified that genotypic differences existed as in the 2015 data. These included the Poisson, negative binomial, zero-inflated, and hurdle models as well as those models that use a transformed response variable. Table 2 summarizes the parameterization of each of the models. The relevant fit statistics included the AICc, BIC, χ^2^/d.f., and variance estimates. Models were selected based on lowest values for the AICc and BIC fit statistics. However, comparing fit statistics is only appropriate between similar models (i.e. Poisson with NB or hurdle models). Additionally, the transformed data sets give variance estimates on a scale not compatible with the GLMM models, making direct comparisons difficult.

Comparing models within the 2015 dataset showed variation between the relevant fit statistics (Table S5). Next the Pearson χ^2^/d.f. value was examined for the Poisson and negative binomial models. For the Poisson model, this value was 4.48, indicating over-dispersion. For the negative binomial model, the observed value of 0.98 could be considered acceptable as it is close to one^15^. As such, it was determined that the remaining models tested would utilize a negative binomial distribution as the basis. The negative binomial GLMM had an AICc of 1936, while the HNB was slightly worse (AICc = 1960). When zero-modified and hurdle models included inflation parameters for each genotype, as opposed to an overall inflation parameter (multiple pi versus single pi), the estimates of count and inflation parameters were sometimes non-estimable. This result was a function of over-parameterization of the model.

### Parameter estimates of genotype effects and inflation probabilities within years

For each count model tested for the 2015 data, estimates of seed count for each of the genotypes were obtained (Table 5). In the models examined, the Type III tests for genotypic differences in the model were significant, indicating that the estimated seed count differences among genotypes differed due to genotypic response to the CHA treatment, confirming the conclusions from the logit and probit model analyses.

**Table 5:** Estimates of seed counts for each genotype in 2015 comparing negative binomial models.

Entry	Name	Hurdle Negative Binomial (HNB) with genotype-specific inflation probabilities			Hurdle Negative Binomial (HNB) with one overall inflation probability			Negative Binomial		
		Mean	95% Confidence Intervals		Mean	95% Confidence Intervals		Mean	95% Confidence Intervals	
1	Freeman	5.466	3.008	9.933	5.441	2.422	12.225	1.993	0.577	6.889
2	Goodstreak	4.266	2.471	7.366	4.250	1.806	9.999	2.356	0.642	8.645
4	LCH13NEDH-11-24	2.557	1.572	4.158	2.562	1.089	6.026	2.080	0.555	7.795
5	NE07531	4.315	2.715	6.859	4.300	1.977	9.354	1.834	0.557	6.038
6	NE09517-1	8.060	4.468	14.538	8.062	2.842	22.864	3.531	0.920	13.546
7	Ruth	4.725	2.649	8.428	4.713	2.239	9.922	1.880	0.544	6.498
8	NE10683	8.502	5.117	14.125	8.493	4.724	15.269	4.625	1.438	14.872
9	Overland	4.142	2.440	7.030	4.111	2.195	7.703	2.217	0.625	7.865
10	Panhandle	5.161	2.735	9.738	5.137	2.347	11.246	2.210	0.607	8.048
11	PSB13NEDH-15-58W	7.510	4.561	12.367	7.499	4.401	12.778	2.957	0.960	9.105
12	Robidoux	8.053	4.849	13.373	8.049	3.671	17.648	3.319	1.008	10.933
13	Settler CL	13.166	6.649	26.070	13.032	5.457	31.125	9.987	2.685	37.143
14	TX09D1172	9.246	5.756	14.854	9.214	5.156	16.467	4.880	1.581	15.067
15	TX10D2063	7.782	4.458	13.583	7.741	2.919	20.531	2.906	0.785	10.755
16	TX10D2230	11.113	6.708	18.410	11.081	6.257	19.626	5.149	1.613	16.435
17	TX10D2363	7.229	4.297	12.159	7.203	3.870	13.406	2.534	0.809	7.942
18	TX11D3008	7.498	4.555	12.342	7.488	4.148	13.517	4.375	1.286	14.887
19	TX11D3026	12.435	7.567	20.434	12.411	7.225	21.319	5.805	1.806	18.662
20	TX11D3049	3.691	2.247	6.064	3.667	1.713	7.849	1.222	0.372	4.019
21	TX11D3112	7.436	4.301	12.857	7.435	3.597	15.366	3.372	0.960	11.851
22	TX11D3129	16.109	8.692	29.855	16.060	7.332	35.178	6.268	1.724	22.795
23	TX12M4004	8.780	4.732	16.292	8.773	3.781	20.355	4.880	1.169	20.379
24	TX12M4063	25.749	14.019	47.293	25.502	12.475	52.136	9.031	2.751	29.650
25	TX12M4065	15.721	9.601	25.744	15.650	8.556	28.626	11.932	3.754	37.922
26	Wesley	7.568	3.740	15.316	7.534	2.458	23.086	1.783	0.450	7.060
27	NE10478-1	9.017	5.360	15.168	9.019	4.867	16.714	4.274	1.342	13.617

The hurdle negative binomial model assuming genotype-specific inflation probabilities performed well on the 2015 data. Of the lines tested in 2015, six had an estimated seed count (if not totally sterile) of five seeds or fewer, with the best line being ‘LCH13NEDH-11-24’ (2.6 seeds per head). The worst lines were ‘TX12M4065’, ‘TX11D3129’ and ‘TX12M4063’ with counts of 15.7, 16.1, and 25.5 seeds, respectively (Table 5). Though the logit and probit models indicated that 2016 showed adequate sterility across most genotypes, hurdle and zero-inflated negative binomial models were also tested to compare estimates between years. During the 2016 trial, the inflation probabilities tended to be higher, so there was more variability around the estimates of seed count than in 2015. As a result, the confidence intervals were larger. However, 17 of the 26 lines had an estimated seed count of less than five seeds. In 2016, TX12M4065 was among this group, indicating that there was an interaction between the year and the genotype that allowed TX12M4065 to be better sterilized. The worst genotypes in 2016 were ‘TX11D3008’ (22.2 seeds) and ‘Ruth’ (26.9 seeds) (Table S8). The differences between years indicated that the CHA may need to be optimized for the growing conditions as well as the genotype when used on a commercial scale. In addition, some genotypes such as LCH13NEDH-11-24, with estimates between 2.5 and 3 in both years, showed stability across differing environmental conditions.

When zero-inflated and hurdle models are run assuming a separate inflation probability for each genotype, the π values provide an estimate of the proportions of zeroes that are in excess of what is expected for a Poisson or negative binomial distribution (Tables 6 and S7). The fit statistics for zero-inflated and hurdle models considering an overall rather than genotype-specific inflation probability may slightly better and did not have issues with over-parameterization. However, there are advantages to examining genotype-specific inflation probabilities, so results from both approaches are examined. These advantages include allowing to compare inflation probabilities between genotypes and determine if there are differences in the frequency of excess zeros. For example, in 2015, TX12M4065 had a low inflation probability of 0.119 and a high estimated seed count, whereas another line with a high estimate seed count (TX12M4063) had a much higher inflation probability of 0.532.

**Table 6:** Estimates of inflation probabilities for each genotype in 2015 with the hurdle negative binomial model.

Genotype	Entry	Estimate	95% Confidence Intervals	
Freeman	1	0.384	0.263	0.522
Goodstreak	2	0.463	0.355	0.575
LCH13NEDH1124	4	0.306	0.186	0.463
NE07531	5	0.444	0.336	0.557
NE095171	6	0.446	0.333	0.564
Ruth	7	0.500	0.401	0.599
NE10683	8	0.411	0.299	0.535
Overland	9	0.167	0.076	0.334
Panhandle	10	0.363	0.241	0.504
PSB13NEDH1558W	11	0.380	0.267	0.508
Robidoux	12	0.333	0.215	0.475
Settler CL	13	0.668	0.518	0.790
TX09D1172	14	0.286	0.178	0.426
TX10D2063	15	0.400	0.278	0.535
TX10D2230	16	0.353	0.238	0.489
TX10D2363	17	0.498	0.402	0.595
TX11D3008	18	0.230	0.123	0.389
TX11D3026	19	0.312	0.195	0.459
TX11D3049	20	0.498	0.403	0.593
TX11D3112	21	0.332	0.210	0.482
TX11D3129	22	0.400	0.280	0.533
TX12M4004	23	0.280	0.155	0.451
TX12M4063	24	0.532	0.423	0.636
TX12M4065	25	0.119	0.048	0.264
Wesley	26	0.663	0.512	0.787
NE104781	27	0.334	0.218	0.474

All estimates were significant at α = 0.05 against a null hypothesis assuming no zero-inflation.

### Simulation experiments

If data such as these counts arise from a process with zero-inflation, it is helpful to know if the zero-inflated models can capture that process well. It is also critical to determine how much information and precision is lost when using models that do not account for the excess zeros. Thus, a dataset known or designed to be zero-inflated can be used to show how much is lost if one uses other modeling strategies. To demonstrate this, five hundred experiments were simulated two times from a zero-inflated process, and the models used to test the experimental crossing block data were used on each of the simulated datasets to monitor overall trends of fit. 95% confidence intervals for the count estimates were constructed on the same scale as the original data.

Empirical coverage probability was calculated as the proportion of confidence intervals that included the true mean of the count for that genotype. These averages are presented in Table 7. Empirical coverage probability ranged from 0% for the logarithmically transformed data to over 90% for the zero-inflated negative binomial models. Hurdle negative binomials had empirical coverage probability around 20%. For the logarithmic, square root, and exponential transformations, the standard errors were also calculated according to the delta method to account for standard errors on the same scale as the data. For these estimates, the empirical coverage probability was always near zero.

**Table 7:** Estimated coverage probabilities for simulated data of a zero-inflated negative binomial distribution under each mixed model.

Model	Coverage	Std Error Coverage
Gaussian	0.089	0.285
LT^a^	0.0002	0.013
ST^b^	0.003	0.052
ET^c^	0.005	0.069
Poisson	0.001	0.032
Negative Binomial	0.525	0.499
HNB^d^	0.199	0.399
ZINB^e^	0.920	0.277

500 simulated datasets were created and evaluated.

^a^LT, model using log-transformed response variable; ^b^ST, model using square-root transformation of the response variable; ^c^ET, model using an exponentially transformed response variable; ^d^HNB, Hurdle Negative Binomial; ^e^ZINB, Zero-inflated negative binomial.

## Discussion

We collected seed count data to determine the frequency of sterility for CHA-treated wheat. The initial data indicated a mean of 5.7 seeds head^−1^ in 2015 and 2.6 seeds head^−1^ in 2016. For most wheat in the Great Plains, the average head of wheat has 30 seeds^27^. Using these estimates, the combined sterility across the genotypes tested was greater than 80% in both years. Researchers in hybrid wheat have not yet set a rule regarding the proportions of sterility required for producing hybrid wheat seed for testing, but it is anticipated that it will be around 75%. Our target number of selfed seeds per head would therefore be seven seeds. As such, this was the cutoff point set for our logit and probit models. Certified hybrid seed for small grains produced using chemical hybridization must be 95% pure hybrid with a maximum of 5% of self-fertilized female parent seed^28^. Under a requirement of 95% hybrid purity, the maximum number of selfed seeds per head is 1–2. However, at the first stages of a breeding program and experimental hybrid testing, the application of the CHA will not be optimized to the female genotype as in commercial seed production; hence a wider range of selfed seed values would be expected from the experimental hybrid crossing block. In addition, few studies have specifically examined the seed count and relative sterility after CHA application, making this approach novel and timely for plant breeders and seed production scientists.

Seed counts were generally lower in 2016 than in 2015, despite keeping treatment rates the same and treatment conditions as similar as possible between the two years. Weather affects the efficacy of the CHA^5^. Both years had higher than normal precipitation for Eastern Nebraska, but at different times. In 2016, the weather was warmer following CHA treatment and may have led to higher sterility. Also, 2015 was the first year of testing with the CHA and due to heavy rainfall in 2015, heads may have been covered later than what would be optimal due to above-average rain at flowering. This delay would have meant the plant may already have been cross-pollinated and counts were thus biased upwards. The combination of more optimal weather conditions and increased familiarity with using the CHA provided overall better sterility in 2016 than in 2015.

The next step was to test if genotypes differed in seed count as a result of the CHA application. The first way this was examined was using logit and probit models and a threshold of seven seeds as a ‘success’ for the binary response variable. The 2015 data showed that there was variation for the frequency of successes as a function of genotype, whereas there were no significant differences among genotypes in 2016. Because the overall sterility was better in 2016, either a lower value could be set to define successes and failures and the models re-tested, or the conclusion could be made that there was high enough sterility in that environment to be confident in the hybrid purity of the seed produced. The mean frequency of meeting the threshold in 2016 across all genotypes was over 0.7, indicating that adequate sterility was achieved at least 70% of the time. In 2015, due to the differences in sterility among genotypes, further evaluation was warranted to better understand those genotypes that were not adequately sterilized. Obtaining more detailed information directly relating to the estimated seed count then became more useful for 2015 and count-based models provided the best information from a biological perspective. The estimates of seed count from the count models can be used in downstream analyses to determine which genotypes are better suited to chemical hybridization if hybrid performance is similar.

Using a number of statistical models, we measured estimated genotypic differences to CHA sterilization on the count of seeds directly. A standard approach in agronomic research to data that are not normally distributed is to apply a variance-stabilizing transformation to the data. The analysis then uses the transformed response variable and assumes it to be normally distributed^11^. The data in this study showed evidence of over-dispersion, and after transformation the variance was not well-controlled. In addition, data transformation in this data set resulted in some values of negative seed count, which is nonsensical, and in all cases complicated the estimation of standard errors or confidence intervals. Using the practical constraint that says that negative estimates of seed count are not appropriate or possible, the results from analyses with data transformation could be considered irrelevant even if the fit statistics are better^18^. In addition, the use of generalized linear mixed models to model the data directly is preferred as estimates are on the same scale as the data. Thus, the best approach was to use models with a Poisson or negative binomial distribution that allow for modeling the counts directly.

The Pearson fit statistic indicated that the Poisson model had issues with over-dispersion. For biological count data, it is common to have variance that exceeds the mean and thus the use of negative binomial models are common in fields like agronomy and biology^11^. The next step was to see how the negative binomial model performed when accounting for excess zeros. The zero-inflated and hurdle models sometimes had poorer fit compared to their standard Poisson or negative binomial GLMM counterparts for each year of testing. The poorer fit may derive from the “small n problem” in which the number of observations from bagged heads that were present for collection for each genotype were too small to obtain good estimates^14^. However, it makes sense that the data would arise from a zero-inflated- or hurdle-type process from a biological perspective.

The zero-inflated and hurdle models provided additional information compared to the other models. Because these models separate counts of zero explicitly from the negative binomial process, estimates of the inflation probability can be obtained as well as estimates of the number of seeds expected for each genotype. Obtaining the inflation probability that can reflect the excess number of zeros from the count if the head is only partially sterilized is helpful for researchers in hybrid wheat. The estimates of π and seed count might assist hybrid wheat breeders in selecting genotypes to use as seed (female) parents based on the consistency of sterilization when using a CHA. Genotypes that have higher inflation probabilities and lower estimates of seed count are preferred in that they will produce purer hybrids. The estimates of seed count can also be used in the analysis of the hybrid yield trials. Because the seed count data are collected the year prior to the planting of the hybrid seed, the estimates of seed count could be helpful in explaining differences in vigor or uniformity of the hybrid plots.

For manufacturing process analysis, results from zero-inflated models were shown to be more conservative in their estimates than directly applying Poisson or Negative Binomial GLMMs, thus lowering Type I error^21^. Thus it was unsurprising that confidence intervals for the estimates of seed counts from zero-inflated and hurdle models were wider than those from the Poisson and Negative Binomial models. This represents a decrease in precision of the estimates, but simulations indicated that use of the wrong model on data with excess zeros gave confidence intervals that did not include the true value of the parameter, and hence were less accurate. The simulations showed that models such as the Poisson and Negative Binomial GLMMs have limits if data shows zero-inflation. For the Poisson and Negative Binomial models, the 95% confidence intervals only contained the true means up to 53% of the time (Table 7). For the zero-inflated models, these values were above 90% while controlling for Type I error. For the transformed data, confidence interval coverage was unreliable even when using the delta method to construct confidence intervals with empirical coverage proportions sometimes as low as zero. This research further indicates that for data that arises from a zero-inflated process, it is best to model that reality appropriately. If coverage is low and Type I error is not well-controlled, standard errors often underestimate the actual variability of the data. However, for zero-inflated and hurdle models, the standard errors are calculated based on the Poisson or negative binomial process, thus making them more accurate. The transformed data models have much poorer coverage values compared to the appropriate count models because the standard errors are too small to provide adequate confidence intervals and Type I error is not controlled. In the Gaussian case, the variance is independent of the mean. In contrast, the variance of Poisson and negative binomial models are directly related to the mean^15^. The best models, therefore, can be evaluated by their ability to accurately estimate variability in addition to targeting the true mean. An initial surprise was the poor performance of the hurdle negative binomial model on the data. However, because the data were generated using a true zero-inflated approach, wherein the zeros could arise from a situation in which zero seeds were observed both as a result of natural processes and use of the CHA, this result is less unexpected. It highlights the importance of incorporating the biology of the data with the statistical strategy: having the models reflect the actual process by which the data arose.

Overall, the confidence intervals for the zero-inflated models, both with experimental and simulated data, are wider than those for other models. However, the simulations indicate that these intervals are much more reliable in covering the true means than for either the transformed data models or the standard Poisson or negative binomial models. What is lost in precision (control of Type I error) is made up for in confidence of the estimates.

In conclusion, the biological complexity of count data from experiments like these allow agronomists to rethink how they analyze data. Including a logit or probit model as a first step can provide guidance to researchers on the need for further evaluation of models and to see if additional useful information can be gained using mixture distribution approaches including zero-inflated and hurdle negative binomial models. Successful implementation of models that account for excess zeros is well-established in medicine, engineering, and ecology. Integrating it into agronomy and plant breeding research is a natural progression.

The estimates from the zero-inflated and hurdle models on the seed count data allowed for separation of genotypes in which the estimate differed by a count of ten seeds or more. As with many experiments, increasing the number of observations enhances the power. The authors recommend that the number of heads isolated to check for sterility are increased, ideally to 10 bags per plot and methods to mechanize the counting process could also play a role in decreasing the cost and time of this assay. Production of hybrid seed on larger scales (i.e. when preparing for advanced yield trials and in commercial settings) may require a different sampling strategy to enhance precision and account for greater spatial variability. Increased observations in the future will provide more information on variability and will decrease the width of confidence intervals.

This research also helps establish best practices for the use of CHAs, establish adequate methods for testing the success of CHA applications, and set appropriate counts of seed to achieve adequate sterility. This work demonstrated that logit and probit models are useful in establishing if a CHA worked as expected. When logit and probit models indicated that there may be genotypic differences, zero-inflated Poisson and zero-inflated negative binomial models provided additional information about the response of a genotype to the CHA. The levels of sterility have broad implications in the study of hybrids produced using chemical hybridization methods, and the ability to demonstrate differences is crucial to researchers producing and evaluating these hybrids. There is little research in the literature examining the relationship between genetics and CHA use in developing hybrids making this work novel and instrumental in planning the future of hybrid wheat breeding.

## Supplementary information

Supplementary Tables and Figures.

Dataset 1.

## Data Availability

Scripts used to evaluate the various models as well as the simulations and data are available at https://github.com/aceasterly/WheatSterility.
