# Supplementary material for: Determining the Efficacy of a Hybridizing Agent in Wheat (Triticum aestivum L.)
Source: Sci Rep. 2019 Dec 27;9:20173. doi: 10.1038/s41598-019-56664-9 (PMC6934762; doi:10.1038/s41598-019-56664-9)
Supplement: Supplementary file 1 — Supplementary Tables and Figures. [file 41598_2019_56664_MOESM1_ESM.docx]

Determining the Efficacy of a Hybridizing Agent in Wheat (*Triticum aestivum* L.­)

Amanda C. Easterly^1^, Walter W. Stroup^2^, Nicholas Garst^1^, Vikas Belamkar^1^, Jean-Benoit Sarazin^3^, Thierry Moittié^3^, Amir M.H. Ibrahim^4^, Jackie C. Rudd^5^, Edward Souza^6^, P. Stephen Baenziger^1^

^1^Department of Agronomy and Horticulture, University of Nebraska, Lincoln, NE 68583-0915, United States

^2^Department of Statistics, University of Nebraska, Lincoln, NE 68583-0963, United States

^3^Asur Plant Breeding, Estrées-Saint-Denis, Picardy, France

^4^Department of Soil and Crop Sciences, Texas A&M University, College Station, TX 77843, United States

^5^Texas AgriLife Research and Extension Center at Amarillo, Amarillo, TX 79106, United States

^6^BASF, Beaver Crossing, NE 68313, United States

Corresponding Author: Amanda C. Easterly, amanda.c.easterly@gmail.com

| **Table S1.** Mean dates of 50% gape in the female parents in 2016. While the genotypes all staged at similar times, their rate of floral development changed later in the season, and Texas germplasm was on average earlier than Nebraska germplasm. | | | | |
| --- | --- | --- | --- | --- |
| Entry | Name | Origin | Mean Gape Date 2015 | Mean Gape Date 2016 |
| 1 | Freeman | NE | 143.4 | 141.8 |
| 2 | Goodstreak | NE | 147.6 | 146.3 |
| 3 | Harry | NE | NA | 146.9 |
| 4 | LCH13NEDH-11-24 | NE | 152.4 | 147.2 |
| 5 | NE07531 | NE | 142.5 | 143.3 |
| 6 | NE09517-1 | NE | 145.4 | 144.1 |
| 7 | Ruth | NE | 152.6 | 144.7 |
| 8 | NE10683 | NE | 147.6 | 144.5 |
| 9 | Overland | NE | 148.6 | 146.6 |
| 10 | Panhandle | NE | 146.6 | 145.8 |
| 11 | PSB13NEDH-15-58W | NE | 150.6 | 144.6 |
| 12 | Robidoux | NE | 144.6 | 144.1 |
| 13 | Settler CL | NE | 143.5 | 143.9 |
| 14 | TX09D1172 | TX | 143.5 | 143.2 |
| 15 | TX10D2063 | TX | 150.1 | 141.1 |
| 16 | TX10D2230 | TX | 143.0 | 140.8 |
| 17 | TX10D2363 | TX | 148.2 | 139.7 |
| 18 | TX11D3008 | TX | 149.7 | 142.1 |
| 19 | TX11D3026 | TX | 144.4 | 143.2 |
| 20 | TX11D3049 | TX | 143.4 | 142.8 |
| 21 | TX11D3112 | TX | 141.6 | 140.4 |
| 22 | TX11D3129 | TX | 146.2 | 143.1 |
| 23 | TX12M4004 | TX | 148.6 | 142.6 |
| 24 | TX12M4063 | TX | 149.5 | 141.7 |
| 25 | TX12M4065 | TX | 145.4 | 143.7 |
| 26 | Wesley | NE | 152.0 | 144.6 |


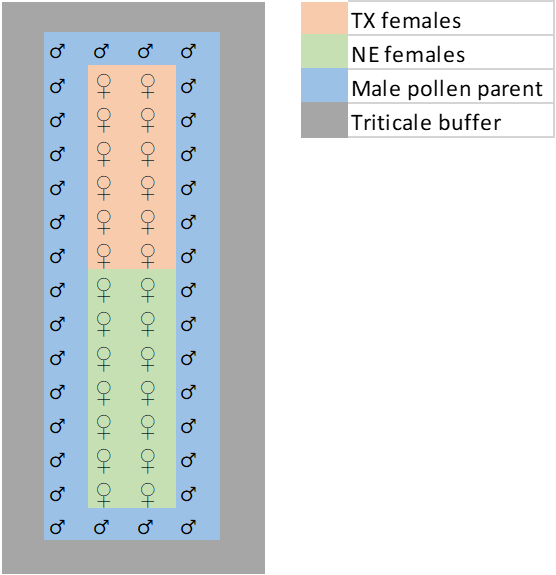


**Figure S1.** Diagram of Hybrid Crossing Block Design. Female parents were randomized within each replication, but placed so that NE and TX lines could be treated with the CHA independently if needed. Male pollen parent was planted to surround the female plots. A triticale buffer was used around each block to prevent pollen contamination from adjacent blocks.

| **Table S2.** Model fit results from the logit and probit models.  Logit and probit models were tested using a threshold of 7 or fewer seeds as a ‘successful’ sterilization. | | | | |
| --- | --- | --- | --- | --- |
| **Year** | **Threshold Model** | **-2 Log Likelihood** | **AICc** | **BIC** |
| 2015 | Logit | 349.43 | 405.43 | 388.24 |
|  | Probit | 349.07 | 409.81 | 387.88 |
| 2016 | Logit | 8.50 | 77.95 | 48.70 |
|  | Probit | 8.06 | 77.51 | 48.27 |

| **Table S3. Genotypic means of probability in 2015 using probit model**  There were significant differences among genotypes in 2015 for logit and probit models assuming successful sterility with a count of seven seeds or fewer. Thus, it was necessary to follow up results by modeling seed count directly. | | | | | | | | | |  |
| --- | --- | --- | --- | --- | --- | --- | --- | --- | --- | --- |
| Entry | Name | t Value | Pr > \|t\| | | Mean Probability | Standard Error | 95% Confidence Interval | | |  |
| 1 | Freeman | 2.25 | 0.03 | | 0.93 | 0.09 | 0.57 | 1.00 | |  |
| 2 | Goodstreak | 2.31 | 0.02 | | 0.96 | 0.06 | 0.60 | 1.00 | |  |
| 4 | LCH13NEDH-11-24 | 0.83 | 0.41 | | 1.00 | 0.01 | 0.00 | 1.00 | |  |
| 5 | NE07531 | 2.21 | 0.03 | | 0.92 | 0.09 | 0.56 | 1.00 | |  |
| 6 | NE09517-1 | 0.66 | 0.51 | | 0.99 | 0.06 | 0.00 | 1.00 | |  |
| 7 | Ruth | 4.61 | 0.00 | | 1.00 | 0.00 | 0.97 | 1.00 | |  |
| 8 | NE10683 | 1.52 | 0.13 | | 0.83 | 0.16 | 0.38 | 0.99 | |  |
| 9 | Overland | 2.20 | 0.03 | | 0.95 | 0.08 | 0.56 | 1.00 | |  |
| 10 | Panhandle | 1.88 | 0.06 | | 0.91 | 0.11 | 0.47 | 1.00 | |  |
| 11 | PSB13NEDH-15-58W | 2.13 | 0.04 | | 0.90 | 0.10 | 0.53 | 0.99 | |  |
| 12 | Robidoux | 1.98 | 0.05 | | 0.90 | 0.11 | 0.50 | 0.99 | |  |
| 13 | Settler CL | 1.62 | 0.11 | | 0.89 | 0.14 | 0.39 | 1.00 | |  |
| 14 | TX09D1172 | 1.67 | 0.10 | | 0.85 | 0.14 | 0.42 | 0.99 | |  |
| 15 | TX10D2063 | 1.32 | 0.19 | | 0.81 | 0.18 | 0.33 | 0.99 | |  |
| 16 | TX10D2230 | 0.58 | 0.56 | | 0.64 | 0.23 | 0.19 | 0.94 | |  |
| 17 | TX10D2363 | 1.75 | 0.08 | | 0.86 | 0.14 | 0.44 | 0.99 | |  |
| 18 | TX11D3008 | 0.36 | 0.72 | | 0.59 | 0.25 | 0.15 | 0.93 | |  |
| 19 | TX11D3026 | 0.84 | 0.41 | | 0.70 | 0.21 | 0.24 | 0.96 | |  |
| 20 | TX11D3049 | 2.53 | 0.01 | | 0.98 | 0.04 | 0.66 | 1.00 | |  |
| 21 | TX11D3112 | 1.38 | 0.17 | | 0.81 | 0.17 | 0.35 | 0.98 | |  |
| 22 | TX11D3129 | 0.27 | 0.79 | | 0.57 | 0.25 | 0.13 | 0.93 | |  |
| 23 | TX12M4004 | 1.23 | 0.22 | | 0.83 | 0.20 | 0.27 | 0.99 | |  |
| 24 | TX12M4063 | 0.59 | 0.56 | | 0.64 | 0.23 | 0.19 | 0.94 | |  |
| 25 | TX12M4065 | -0.77 | 0.44 | | 0.32 | 0.21 | 0.05 | 0.77 | |  |
| 26 | Wesley | 1.86 | 0.07 | | 0.93 | 0.11 | 0.46 | 1.00 | |  |
| 27 | NE10478-1 | 1.16 | 0.25 | | 0.76 | 0.19 | 0.31 | 0.97 | |  |
| **Table S4. Genotypic means of probability in 2016 using the logit model.**  There were not significant differences among genotypes in 2016 using logit and probit models, so these results were of limited use, other than to confirm that the means were generally high, indicating that most observations were sterilized and had seven seeds or fewer per head. | | | | | | | | | | |
| Entry | Name | t Value | | Pr > \|t\| | Mean Probability | Standard Error | 95% Confidence Interval | | | |
| 1 | Freeman | 0.00 | | 1.00 | 1.00 | 0.03 | 0.00 | | 1.00 | |
| 2 | Goodstreak | 0.88 | | 0.39 | 0.72 | 0.22 | 0.23 | | 0.96 | |
| 3 | Harry | 0.00 | | 1.00 | 1.00 | 0.03 | 0.00 | | 1.00 | |
| 4 | LCH13NEDH-11-24 | 0.00 | | 1.00 | 1.00 | 0.03 | 0.00 | | 1.00 | |
| 5 | NE07531 | 0.12 | | 0.91 | 1.00 | 0.00 | 0.00 | | 1.00 | |
| 6 | NE09517-1 | 0.00 | | 1.00 | 1.00 | 0.03 | 0.00 | | 1.00 | |
| 7 | Ruth | 0.12 | | 0.90 | 1.00 | 0.00 | 0.00 | | 1.00 | |
| 8 | NE10683 | 0.00 | | 1.00 | 1.00 | 0.03 | 0.00 | | 1.00 | |
| 9 | Overland | 0.08 | | 0.93 | 1.00 | 0.02 | 0.00 | | 1.00 | |
| 10 | Panhandle | 0.01 | | 0.99 | 1.00 | 0.03 | 0.00 | | 1.00 | |
| 11 | PSB13NEDH-15-58W | 1.87 | | 0.07 | 0.84 | 0.12 | 0.47 | | 0.97 | |
| 12 | Robidoux | 0.12 | | 0.91 | 1.00 | 0.00 | 0.00 | | 1.00 | |
| 13 | Settler CL | 0.00 | | 1.00 | 1.00 | 0.03 | 0.00 | | 1.00 | |
| 14 | TX09D1172 | 0.14 | | 0.89 | 1.00 | 0.00 | 0.00 | | 1.00 | |
| 15 | TX10D2063 | 2.55 | | 0.01 | 0.92 | 0.07 | 0.62 | | 0.99 | |
| 16 | TX10D2230 | 1.26 | | 0.21 | 0.74 | 0.16 | 0.34 | | 0.94 | |
| 17 | TX10D2363 | -0.08 | | 0.94 | 0.00 | 0.03 | 0.00 | | 1.00 | |
| 18 | TX11D3008 | 1.83 | | 0.08 | 0.84 | 0.12 | 0.46 | | 0.97 | |
| 19 | TX11D3026 | 0.13 | | 0.90 | 1.00 | 0.00 | 0.00 | | 1.00 | |
| 20 | TX11D3049 | 0.13 | | 0.90 | 1.00 | 0.00 | 0.00 | | 1.00 | |
| 21 | TX11D3112 | 1.62 | | 0.11 | 0.80 | 0.14 | 0.41 | | 0.96 | |
| 22 | TX11D3129 | -0.10 | | 0.92 | 0.48 | 0.20 | 0.15 | | 0.82 | |
| 23 | TX12M4004 | 0.12 | | 0.90 | 1.00 | 0.00 | 0.00 | | 1.00 | |
| 24 | TX12M4063 | 2.73 | | 0.01 | 0.94 | 0.05 | 0.68 | | 0.99 | |
| 25 | TX12M4065 | 1.76 | | 0.09 | 0.88 | 0.12 | 0.42 | | 0.99 | |
| 26 | Wesley | 1.03 | | 0.31 | 0.80 | 0.22 | 0.21 | | 0.98 | |

| **Table S5. Model fit results for 2015 sterility analysis.**  Best models are determined based on lowest values of Akaike’s Information Criterion, corrected for small sample sizes (AICc) and Bayesian Information Criterion (BIC) as well as a value of χ^2^/d.f. that is close to 1 for the negative binomial model. In addition, best models provide estimates of genotypic means that make sense on the data scale (i.e. are greater than or equal to zero). | | | | |
| --- | --- | --- | --- | --- |
| **Model** | **-2 Log Likelihood** | **AICc** | **BIC** | **χ^2^/d.f.** |
| Gaussian (Normal) | 2416.65 | 2422.72 | 2420.81 | 48.64 |
| Poisson | 2982.39 | 3043.39 | 3021.21 | 4.48^a^ |
| Negative Binomial | 1875.49 | 1936.24 | 1914.31 | 0.98^a^ |
| LT^b^ | 1067.19 | 1073.26 | 1071.35 | 0.98 |
| ST^c^ | 1232.55 | 1238.62 | 1236.71 | 1.57 |
| ET^d^ | 1677.45 | 1683.52 | 1681.61 | 5.71 |
| ZINB^e^ (single pi) | 1401.3 | 1466.8 | 1442.9 | NA |
| ZINB (multiple pi) | 1401.3 | 1530.9 | 1477.6 | NA |
| HNB^f^ (single pi) | 1896.0 | 1961.5 | 1937.6 | NA |
| HNB (multiple pi) | 1871.5 | 2001.0 | 1947.7 | NA |
| ^a^Indicates the use of the Pearson Chi-Square/df calculation as opposed to the Generalized Chi-Square/df  ^b^LT, model using log-transformed response variable; ^c^ST, model using square-root transformation of the response variable; ^d^ET, model using an exponentially transformed response variable; ^e^ ZINB, Zero-inflated negative binomial; ^f^ HNB, Hurdle negative binomial  ^g^Chi-Square/df calculation not calculable in PROC NLMIXED | | | | |

| **Table S6. Model fit results for 2016 sterility analysis.**  Best models are determined based on lowest values of Akaike’s Information Criterion, corrected for small sample sizes (AICc) and Bayesian Information Criterion (BIC) as well as a value of χ^2^/d.f. that is close to 1 for the negative binomial model. In addition, best models provide estimates of genotypic means that make sense on the data scale (i.e. are greater than or equal to zero). | | | | |
| --- | --- | --- | --- | --- |
| **Model** | **-2 Log Likelihood** | **AICc** | **BIC** | **χ^2^/d.f.** |
| Gaussian (Normal) | 959.31 | 965.47 | 963.47 | 2967.15 |
| Poisson | 783.06 | 846.88 | 820.49 | 2.17^a^ |
| Negative Binomial | 534.69 | 598.51 | 572.12 | 0.63^a^ |
| LT^b^ | 429.93 | 435.09 | 433.09 | 0.62 |
| ST^c^ | 470.07 | 476.23 | 474.23 | 0.81 |
| ET^d^ | 661.64 | 667.79 | 665.80 | 2.77 |
| ZINB^e^ (single pi) | 333.9 | 406.2 | 375.5 | na^g^ |
| ZINB (multiple pi) | 333.0 | 491.9 | 409.3 | na^g^ |
| HNB^f^ (single pi) | 569.0 | 641.4 | 610.6 | na^g^ |
| HNB (multiple pi) | 522.6 | 681.5 | 598.9 | na^g^ |
| ^a^Indicates the use of the Pearson Chi-Square/df calculation as opposed to the Generalized Chi-Square/df  ^b^LT, model using log-transformed response variable; ^c^ST, model using square-root transformation of the response variable; ^d^ET, model using an exponentially transformed response variable; ^e^ ZINB, Zero-inflated negative binomial; ^f^ HNB, Hurdle negative binomial  ^g^Chi-Square/df calculation not calculable in PROC NLMIXED | | | | |

| **Table S7. Estimates of inflation probabilities for each genotype in 2016 for the hurdle negative binomial model.**  As with Table 10, these data reflect the hurdle negative binomial model which allowed for a specific estimate of each genotype for inflation. | | | | |
| --- | --- | --- | --- | --- |
| Name | Entry | Estimate | 95% Confidence Interval | |
| Freeman | 1 | 0.858 | 0.408 | 0.982 |
| Goodstreak | 2 | 0.336 | 0.042 | 0.852 |
| Harry | 3 | 0.798 | 0.308 | 0.972 |
| LCH13NEDH1124 | 4 | 1.000 | 0.000 | 1.000 |
| NE07531 | 5 | 0.858 | 0.417 | 0.981 |
| NE095171 | 6 | 0.601 | 0.206 | 0.898 |
| Ruth | 7 | 1.000 | 0.000 | 1.000 |
| NE10683 | 8 | 0.665 | 0.161 | 0.954 |
| Overland | 9 | 0.444 | 0.178 | 0.747 |
| Panhandle | 10 | 0.501 | 0.081 | 0.920 |
| PSB13NEDH1558W | 11 | 0.667 | 0.333 | 0.890 |
| Robidoux | 12 | 0.714 | 0.325 | 0.928 |
| Settler CL | 13 | 0.665 | 0.161 | 0.954 |
| TX09D1172 | 14 | 1.000 | 0.000 | 1.000 |
| TX10D2063 | 15 | 0.600 | 0.297 | 0.842 |
| TX10D2230 | 16 | 0.374 | 0.125 | 0.714 |
| TX10D2363 | 17 | 0.000 | 0.000 | 1.000 |
| TX11D3008 | 18 | 0.600 | 0.297 | 0.842 |
| TX11D3026 | 19 | 0.728 | 0.412 | 0.911 |
| TX11D3049 | 20 | 0.777 | 0.417 | 0.944 |
| TX11D3112 | 21 | 0.444 | 0.178 | 0.747 |
| TX11D3129 | 22 | 0.375 | 0.125 | 0.715 |
| TX12M4004 | 23 | 0.749 | 0.374 | 0.937 |
| TX12M4063 | 24 | 0.500 | 0.246 | 0.754 |
| TX124065 | 25 | 0.667 | 0.269 | 0.916 |
| Wesley | 26 | 0.665 | 0.161 | 0.954 |

| **Table S8. Estimates of seed count in 2016 for each genotype using the hurdle negative binomial model with genotype-specific inflation probabilities.** | | | |
| --- | --- | --- | --- |
| Name | Estimate | Lower CI | Upper CI |
| Freeman | 2.507094241 | 0.162349693 | 38.71594356 |
| Goodstreak | 7.673992013 | 3.272162276 | 17.99732056 |
| Harry | 1.422813832 | 0.560128941 | 3.614166403 |
| LCH13NEDH-11-24 | 2.953129202 | 0.448979083 | 19.4240053 |
| NE07531 | 1.561088469 | 0.274362319 | 8.882404914 |
| NE09517-1 | 2.249338239 | 0.742066958 | 6.818148224 |
| NE10683 | 1.974939417 | 0.648682976 | 6.012776421 |
| Overland | 2.356311441 | 0.524481709 | 10.58607671 |
| Panhandle | 4.220995601 | 1.983955827 | 8.980443829 |
| PSB13NEDH-15-58W | 7.092250162 | 3.572831797 | 14.07847198 |
| Robidoux | 3.087861274 | 0.708970371 | 13.44892204 |
| Ruth | 26.87343526 | 9.327983428 | 77.42096975 |
| Settler CL | 2.709239793 | ne^a^ | ne |
| TX09D1172 | 2.951312967 | 0.522669722 | 16.66491834 |
| TX10D2063 | 8.01430205 | 4.241330755 | 15.14360493 |
| TX10D2230 | 2.190825413 | 0.484357725 | 9.909444497 |
| TX10D2363 | 7.001299046 | 1.77898423 | 27.55403196 |
| TX11D3008 | 22.18609713 | 12.19598257 | 40.35943007 |
| TX11D3026 | 11.29920554 | 4.852718898 | 26.30938417 |
| TX11D3049 | 2.601613905 | 0.666773659 | 10.15096325 |
| TX11D3112 | 7.01161745 | 3.659101976 | 13.43574997 |
| TX11D3129 | 5.071810191 | 2.709579743 | 9.493449559 |
| TX12M4004 | 0.778160957 | ne | ne |
| TX12M4063 | 6.422626002 | 3.031548795 | 13.60694732 |
| TX12M4065 | 3.556455365 | 1.271547466 | 9.947229735 |
| Wesley | 1.299861217 | 0.35289942 | 4.787877476 |
| ^a^ne denotes non-estimability, because estimates were less than 1. | | | |

| **Table S9. Genotype parameters used in simulation study.**  25 genotypes were used and were set to have mean seed counts (λ). An overall inflation probability of π=0.35 was used for the “single pi” datasets and genotype-specific inflation probabilities (π) were used to test the situation where there is a different mean inflation probability as well as mean seed count. Data were simulated using a zero-inflated negative binomial process. | | |
| --- | --- | --- |
| Entry | λ (Mean Seed Count) | π (Inflation Probability, genotype-specific) |
| 1 | 6 | 0.15 |
| 2 | 4 | 0.77 |
| 3 | 3 | 0.22 |
| 4 | 4 | 0.01 |
| 5 | 8 | 0.21 |
| 6 | 5 | 0.45 |
| 7 | 9 | 0.01 |
| 8 | 4 | 0.99 |
| 9 | 5 | 0.74 |
| 10 | 8 | 0.51 |
| 11 | 8 | 0.34 |
| 12 | 13 | 0.37 |
| 13 | 10 | 0.35 |
| 14 | 8 | 0.11 |
| 15 | 11 | 0.43 |
| 16 | 7 | 0.63 |
| 17 | 8 | 0.99 |
| 18 | 12 | 0.40 |
| 19 | 4 | 0.28 |
| 20 | 8 | 0.48 |
| 21 | 16 | 0.56 |
| 22 | 9 | 0.63 |
| 23 | 27 | 0.27 |
| 24 | 18 | 0.51 |
| 25 | 8 | 0.33 |

| **Table S10.** Estimated coverage probabilities for zero-inflated negative binomial experiments under each mixed model for second run of simulation^a^ | | |
| --- | --- | --- |
| Model | Coverage | Std Error Coverage |
| Gaussian | 0.084 | 0.278 |
| LT^b^ | 0.0002 | 0.016 |
| ST^c^ | 0.003 | 0.051 |
| ET^d^ | 0.004 | 0.065 |
| Poisson | 0.0007 | 0.0268 |
| Negative Binomial | 0.529 | 0.499 |
| HNB^e^ | 0.170 | 0.379 |
| ZINB^f^ | 0.917 | 0.276 |
| ^a^Each simulated data set was built to contain 500 observations for an overall total of 50,000 observations across the entire simulation. ‘ne’ denotes estimates that were non-estimable.  ^b^LT, model using log-transformed response variable; ^c^ST, model using square-root transformation of the response variable; ^d^ET, model using an exponentially transformed response variable; ^e^ HNB, Hurdle Negative Binomial; ^f^ZINB, Zero-inflated negative binomial | | |
